# Coordination of Caregiver Naming and Children’s Exploration of Solid Objects and Nonsolid Substances

**DOI:** 10.3389/fpsyg.2022.945664

**Published:** 2022-07-05

**Authors:** Lynn K. Perry, Stephanie A. Custode, Regina M. Fasano, Brittney M. Gonzalez, Adriana M. Valtierra

**Affiliations:** ^1^Department of Psychology, University of Miami, Coral Gables, FL, United States; ^2^Bill Wilkerson Center for Otolaryngology and Communication Sciences, Vanderbilt University, Nashville, TN, United States

**Keywords:** caregiver-child interaction, word learning, manual-visual exploration, head cameras, exploration

## Abstract

When a caregiver names objects dominating a child’s view, the association between object and name is unambiguous and children are more likely to learn the object’s name. Children also learn to name things other than solid objects, including nonsolid substances like applesauce. However, it is unknown how caregivers structure linguistic and exploratory experiences with nonsolids to support learning. In this exploratory study of caregivers and children (*n* = 14, 8 girls; *M =* 20.50 months) we compare caregiver-child free-play with novel solid objects and novel nonsolid substances to identify the linguistic and exploratory experiences associated with children’s word learning. We found systematic differences in interactions with novel objects, such that children performed more manual actions on solids than nonsolids and caregivers named solids more than nonsolids. Additionally, there was less synchrony between caregivers’ naming and children’s manual and visual exploration of nonsolids than solids. Consistent with prior work, we found that synchronous naming was associated with accurate recognition of solid object names. However, naming synchrony was not associated with recognition of nonsolid substance names or with generalization. Together these findings, though exploratory, suggest the coordination of caregiver-child play can shape what children remember about novel word-object associations for solid objects, but not nonsolid substances.

## Introduction

Caregivers play a critical role in children’s development by structuring their learning environments. From parental emotional modeling and responsiveness (Denham et al., 1997), to scaffolding problem solving and executive function (Hammond et al., 2012), caregivers provide structure supporting learning. Vygotsky went so far as to propose, “it is through others that we become ourselves,” (Vygotsky, 1987) introducing the idea that social interactions “scaffold” children’s development. No domain exemplifies this idea as well as language development. Although word learning was previously considered to be a problem so rife with ambiguities that it could only be solved *via* innate cognitive constraints (Quine, 1960; Markman, 1991), increasing evidence reveals that interactions with caregivers serve to disambiguate the word learning problem. As they play, children tend to hold objects close to their faces, dominating their view (Smith et al., 2011). When caregivers name objects dominating the child’s view, the association between object and label is unambiguous (Gillette et al., 1999), and children are more likely to learn the object’s name (Yu and Smith, 2012; Yurovsky et al., 2013), potentially because these views provide useful information about object shape—necessary for later recognition (Biederman, 1995) and generalization to other category members (Landau et al., 1988). Children also learn to name and recognize things other than solid objects, including nonsolid substances like applesauce (Rips and Hespos, 2015, 2019), but it is unknown how caregivers structure linguistic and exploratory experiences to support that learning. Here we use head-worn cameras to compare differences in caregiver-child free-play with novel solid objects and novel nonsolid substances to identify the linguistic, visual, and manual experiences associated with children’s recognition and generalization.

### The Role of Word Learning in Object and Substance Recognition

#### Solid Objects and the Development of the Shape Bias

Children are skilled word learners—producing, on average, nearly 700 words by age 2.5 years (Dale and Fenson, 1996). In addition to mapping a word to a referent and remembering that mapping later, successful word learning requires being able to generalize words to new instances of a category. The word *cup* does not just refer to a child’s favorite sippy cup, but also refers to paper coffee cups and glass tumblers. Thus, to learn new words, children acquire word learning biases that help them determine the features that are relevant for category membership. For example, by about 2 years of age, children acquire a bias to generalize the names of novel objects to other objects similar in shape (i.e., the “shape bias;” Landau et al., 1988).

The shape bias emerges from regularities in children’s early noun vocabularies. The majority of early-learned words name solid objects in categories well-organized by similarity in shape (e.g., “ball,” “spoon”; Samuelson and Smith, 1999), making shape an important part of children’s object recognition. Indeed, shape is even integral to many accounts of adults’ object recognition (Biederman, 1995). Longitudinal training studies demonstrate that vocabulary plays a causal role in the development of the shape bias (Samuelson, 2002; Smith et al., 2002; Perry et al., 2010). Individual differences studies reveal that when children have vocabularies that differ from this typical structure, they do not show a shape bias and instead attend to objects’ materials or colors (Perry and Samuelson, 2011; Perry et al., 2016; Perry and Saffran, 2017; Slone and Sandhofer, 2017; Perry and Kucker, 2019). Together the training studies and individual differences work demonstrate that learning a lot of words naming individual categories organized by similarity in shape teaches children to attend to shape in general as they learn to recognize and generalize the names of solid objects. However, it is not clear how they initially learn that shape mattered for each of those individual categories in the first place. Furthermore, it is not clear how children begin to recognize and generalize the names of other types of categories for which shape is not relevant, such as nonsolid substances.

#### Nonsolid Substances and the Development of the Material Bias

Nonsolid substances, such as oatmeal and applesauce, belong to categories organized by similarity in material. Shape is irrelevant for these categories’ membership because nonsolids take on the shape of their containers. Children learn to generalize the names of nonsolid substances by similarity in material (i.e., show a “material bias”). However, that bias is later acquired (Samuelson and Smith, 1999; Subrahmanyam et al., 1999) and is less robust (Samuelson and Horst, 2007; Perry et al., 2014) than the shape bias. This fragility of the early material bias is underscored by the mixed results found across studies. For example, Soja et al. (1991) found that children showed a material bias when the named exemplar shared two properties with the material match test item (same material and color, different shape) and only one property with the shape match test item (same shape, different material and color). Subsequent work by Samuelson and Horst (2007), revealed that children did not show a material bias when the named exemplar only shared one property with the material match test item (same material, different color and shape). Further, they found that the likelihood of children’s showing a material bias for nonsolids is dependent on the specific familiar items used on warm-up trials, the configuration of nonsolid stimuli in pieces versus wholes, and the child’s choices on previous trials. In contrast, the shape bias for solid objects is so strong, in contrast, that experimenters can label solid stimuli with mass syntax (typically reserved for non-individuatable things without a coherent shape), and children will still show a shape bias (Soja, 1992). One salient reason for the differences in acquisition of the shape and material biases is the disproportionate number of words children learn naming solid objects relative to nonsolid substances. By the time children are 30 months old they will, on average, have learned to produce the names of 197 solid objects but only 14 nonsolid substances (Samuelson and Smith, 1999). Additionally, although the solid objects children learn to name come from a variety of superordinate categories (vehicles, small household objects, furniture), 12 of the 14 nonsolids children produce before 30 months are foods or drinks (Fenson et al., 1994) and 2 of the 14 name outside things (rain, water). Thus, although children may have many opportunities to experience additional nonsolid substances or things with ambiguous solidity, such as sand or snow or even their own drool, what they learn about nonsolids and *naming* appears to be relatively constrained in context early in development. An additional, non-mutually exclusive explanation for the difficulty children have in learning the names of nonsolids relative to solids is that nonsolid substances are difficult for the perceptual system to individuate, making it unclear what to associate with a new name. This idea was introduced by Samuelson and Smith (2000) as an extension of Gentner’s natural-partitions hypothesis (see, e.g., Gentner, 1982), that nouns are easier for children to learn than verbs, because they often name concrete objects that are more easily individuated than verbs.

Despite the apparent lack of robust knowledge of nonsolids and naming in toddlerhood, even very young infants are able to visually discriminate solids and nonsolids, suggesting they have formed perceptual predictions about how nonsolids behave (e.g., vanMarle and Scholl, 2003; Hespos et al., 2016; Anderson et al., 2018). For example, when habituated to a scene of a liquid being stirred in a cup, 5-month-old infants will continue to be habituated to a scene in which the liquid is poured out of the cup, but dishabituate to a scene in which the liquid remains in the cup as it is poured (Hespos et al., 2009), suggesting sensitivity to the differences in how solids and nonsolids move. Additionally, 8-month-old infants track how many solid objects are placed behind a screen, but do not track how many piles of sand are poured behind a screen (Huntley-Fenner et al., 2002), suggesting that they are sensitive to differences in the extent to which solids and nonsolids are individuatable and countable. This rich body of literature demonstrates that children have some understanding of the differences between solid objects and nonsolid substances and can visually discriminate them in a general way. However, recognizing that something is nonsolid and therefore behaves differently than something solid is not the same as recognizing it as a specific nonsolid with a specific material—a skill critical to learning to name it. This sort of recognition appears to be influenced by children’s visual and manual exploration.

### The Role of Exploration in Object and Substance Recognition

Children’s early play and manual exploration with solid objects provides them with the multiple views necessary for later recognition (Smith et al., 2011). This exploration teaches them, for example, that objects are three-dimensional, and that objects have backs, even if they cannot be seen (Soska et al., 2010). As children gain more exploration experiences, they learn that some object views are more informative than others and begin to spend more time exploring objects stabilized on the planar view in which the major axis of the object is parallel or perpendicular to the line of sight (Pereira et al., 2010). These views may be particularly relevant for learning about an object’s shape—a necessary feature to recognizing most solid objects. When children’s exploration leads to bouts of sustained attention in which their body stabilizes as they hold an object dominating their view (Bambach et al., 2016), they are also more likely to learn the name of that object (Yu and Smith, 2012; Yu et al., 2019). Together, these data reveal that early manual-visual exploration can teach children how to recognize solid objects and learn their names.

Manual exploration may be especially useful for recognizing nonsolid substances, which do not have a coherent, consistent shape. In general, tactile information is especially necessary even for adults’ recognition of materials (Lederman and Klatzky, 1990). However, because children’s learning about nonsolid substances is constrained to mealtimes, they have limited opportunities to touch and explore most nonsolids. During the mealtime context, in which children typically sit in a highchair, children are able to form the manual actions needed to recognize nonsolids by their material, such as touching, grabbing, and breaking food into pieces before eating. Indeed, when children are placed in a highchair in the laboratory, they show increases in their messy manual exploration on nonsolids relative to when they sit at a typical laboratory table in a booster seat, and are more likely to correctly generalize the names of novel nonsolids to other substances of the same material (Perry et al., 2014). The highchair serves as an early context cue to the types of action patterns necessary for recognition of nonsolids. Eventually, though, kids leave their highchairs, and cannot touch and eat all new nonsolid substances they encounter to learn about their material. How do they learn to visually recognize nonsolid materials by name? How do they learn to associate a new name for a nonsolid substance with its material in order to generalize to new category members? Here we use caregiver-child play with both solids and nonsolids as a window into that initial learning experience.

### The Role of Caregivers in Early Word Learning

Children’s early word learning experiences are shaped by interactions with their caregivers (Bruner, 1975; Carpenter et al., 1998). Many studies reveal that the way in which caregivers name objects matters more for children’s word learning than the number of times that they name them (e.g., Samuelson et al., 2011; Yu and Smith, 2012; Perry et al., 2021). Caregivers will often name and talk about objects that their children are attending to, temporally linking words and referents (Yu and Smith, 2012; Tamis-LeMonda et al., 2014; Golinkoff et al., 2015). Similarly, caregivers tend to spatially segregate referents during play (Samuelson et al., 2011), facilitating children’s linking of words and referents (Benitez and Smith, 2012; Axelsson et al., 2016). Additionally, caregivers use of gestures and attentional cues help guide children’s attention to specific referents (Custode and Tamis-LeMonda, 2020). Finally, caregiver language is highly associated with location and activity (Tamis-LeMonda et al., 2019; Custode and Tamis-LeMonda, 2020), possibly helping children to use context as a cue to meaning, and learn higher order semantic relationships between words as early as they do (Bergelson and Aslin, 2017). Despite the wealth of evidence showing that caregiver talk is structured systematically in ways that facilitate learning, it remains unclear how caregivers might structure learning experiences to facilitate the attention to shape and material needed to recognize and generalize solid objects and nonsolid substances, respectively.

#### Utilizing Head-Worn Cameras to Gain a First-Person Perspective of Exploration

The utilization of head cameras worn during caregiver-child free-play has allowed researchers to examine caregiver-child interactions from a first-person perspective and gain insight into the linguistic, visual, and manual experiences associated with children’s word learning (Smith et al., 2015, 2018; Clerkin et al., 2017; Suanda et al., 2019; McQuillan et al., 2020). This paradigm involves both a caregiver and a child wearing small cameras low on the forehead. Because young children tend to move their eyes and head in synchrony (Yoshida and Smith, 2008), head cameras allow research to capture experience from the child’s perspective. Although previously used to study social gaze and manual exploration as children play with familiar and novel solid objects (Yu and Smith, 2012, 2013; Yurovsky et al., 2013; Chen et al., 2020), head cameras can provide the first-person perspectives needed to gain insight into children’s learning about shape and material as they play with solids and nonsolids.

### Current Study

Although young children visually discriminate solid and nonsolid substances, reflecting an understanding of how these broad classes of things behave, their early understanding of nonsolids and naming appears to be relatively fragile. Missing from prior work is an understanding of the types of experiences children need to learn about specific nonsolid substances and their names. Here we utilize head cameras during caregiver-child free-play to assess how caregivers name and children manually and visually explore novel solid objects and nonsolid substances by analyzing head camera video frame by frame. This approach yields a large number of data points per participant, which allows for meaningful analysis of behavior even from a small number of participants (here *n* = 14 dyads; *cf.* DeBolt et al., 2020) and is consistent with samples used in other head camera studies (e.g., Yu and Smith, 2012; Yurovsky et al., 2013; Suanda et al., 2019). We then explore which linguistic, visual, and manual experiences are associated with children’s subsequent recognition and generalization of novel names at test. In particular, we are interested in (1) how caregivers’ naming events and children’s manual and visual behaviors differ during exploration of solid objects versus nonsolid substances and (2) how caregivers’ naming events, particularly naming events that are synchronous with children’s manual and visual exploration relate to children’s subsequent recognition and generalization accuracy. We hypothesize higher levels of caregiver naming, child exploration, and coordination of these behaviors for solid objects than nonsolid substances because of the disproportionately large number of prior experiences dyads have had naming and exploring solids relative to nonsolids (*cf.* Samuelson, 2002; Perry et al., 2014). Alternatively, if naming and exploration behaviors are equivalent across solids and nonsolids, we nevertheless hypothesize that there will be more synchrony of these behaviors for solids than nonsolids. Finally, we hypothesize that regardless of solidity, increased naming synchrony will be associated with children’s successful recognition and generalization of novel names. To our knowledge, this exploratory study is the first to employ head cameras to examine differences in children’s exploration and learning of solid and nonsolid substances.

## Materials and Methods

### Participants

Fourteen monolingual English learning 1–3-year-old children (*M* = 20.50 months; SD: 5.28 months; range: 14– 34 months; eight girls) and their caregivers participated. Five children were Latinx (3 White, 1 Black, and 1 multiracial) and nine children were non-Latinx (6 White and 3 multiracial). Caregivers reported their education level: 36% had some college or an Associate’s degree, 14% had a Bachelor’s degree, and 50% had a Master’s or Doctoral degree. An additional six children were excluded from data analysis due to fussiness (3) or equipment error (3), such as the camera being pushed up too far on the forehead to see the child’s perspective. Although high, the fussiness rate is not unexpected for a study in which children wear equipment on their head. The study was conducted in accordance with APA ethical standards and was approved by the University Institutional Review Board. Caregivers gave their informed consent and children received a small toy for their participation. Caregivers reported children’s productive vocabulary knowledge on the MacArthur-Bates Communicative Developmental Inventory Words and Sentences Form (MCDI; Fenson et al., 1994). Children’s mean vocabulary size was 139.93 words (*SD =* 132.38, range: 2–364 words).

### Procedure Overview

Participants completed three tasks: (1) *free-play*, to examine the timing and frequency of children’s visual and manual exploration and caregivers’ naming; (2) *recognition*, to assess children’s recognition memory for novel object/substance names introduced during free-play; and (3) *generalization*, to assess children’s ability to extend the novel names to novel objects/substances of the same shape or material.

#### Stimuli

**Figure 1:**
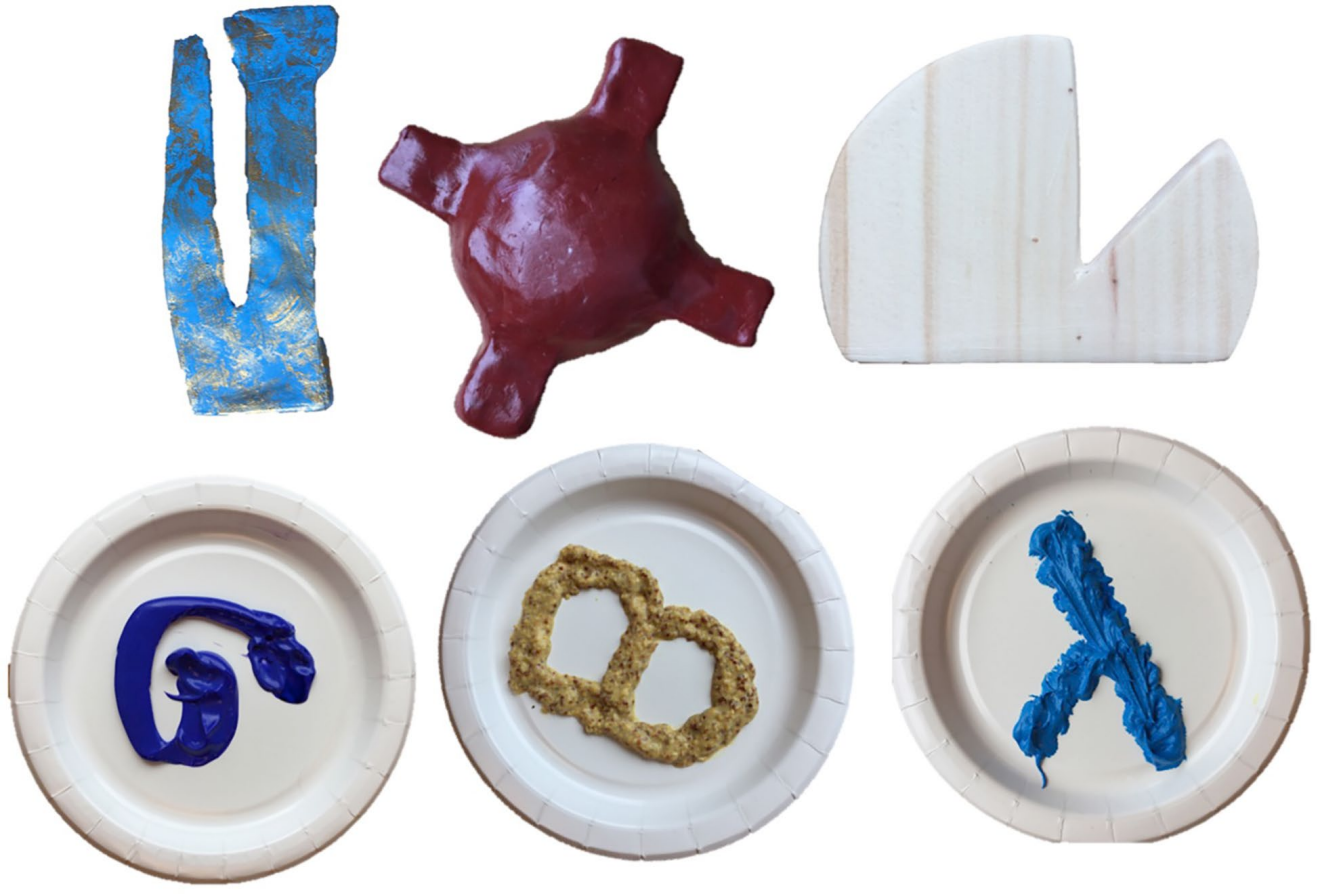
Novel solid object exemplars (top row) and nonsolid substance exemplars (bottom row) used in the free-play, recognition, and generalization tasks. Clockwise from top left, the objects’ names and materials are sebby (textured clay), tulver (clay), blicket (wood), bosa (icing), teema (seed style Dijon mustard), and modi (dyed mayonnaise).

Six novel stimuli (three solids, three nonsolids) were used as exemplars in all tasks (see Figure 1). An additional twelve novel stimuli were designed to match one of the exemplars in shape or material and used in the generalization task. Stimuli were chosen to allow for a variety of colors and textures to be represented. Eight familiar stimuli were used on warm-up trials in the recognition and generalization tasks. These were common objects (e.g., cup, shoe) and substances (e.g., applesauce, oatmeal) that are typically learned before 2 years (Dale and Fenson, 1996). Novel object names (sebby, blicket, tulver, modi, bosa, and teema) were designed to be phonotactically permissible in English. All novel nonsolid substances were edible (e.g., dyed mayonnaise; icing) to allow children to explore freely.^1^

#### Free-Play

**Figure 2:**
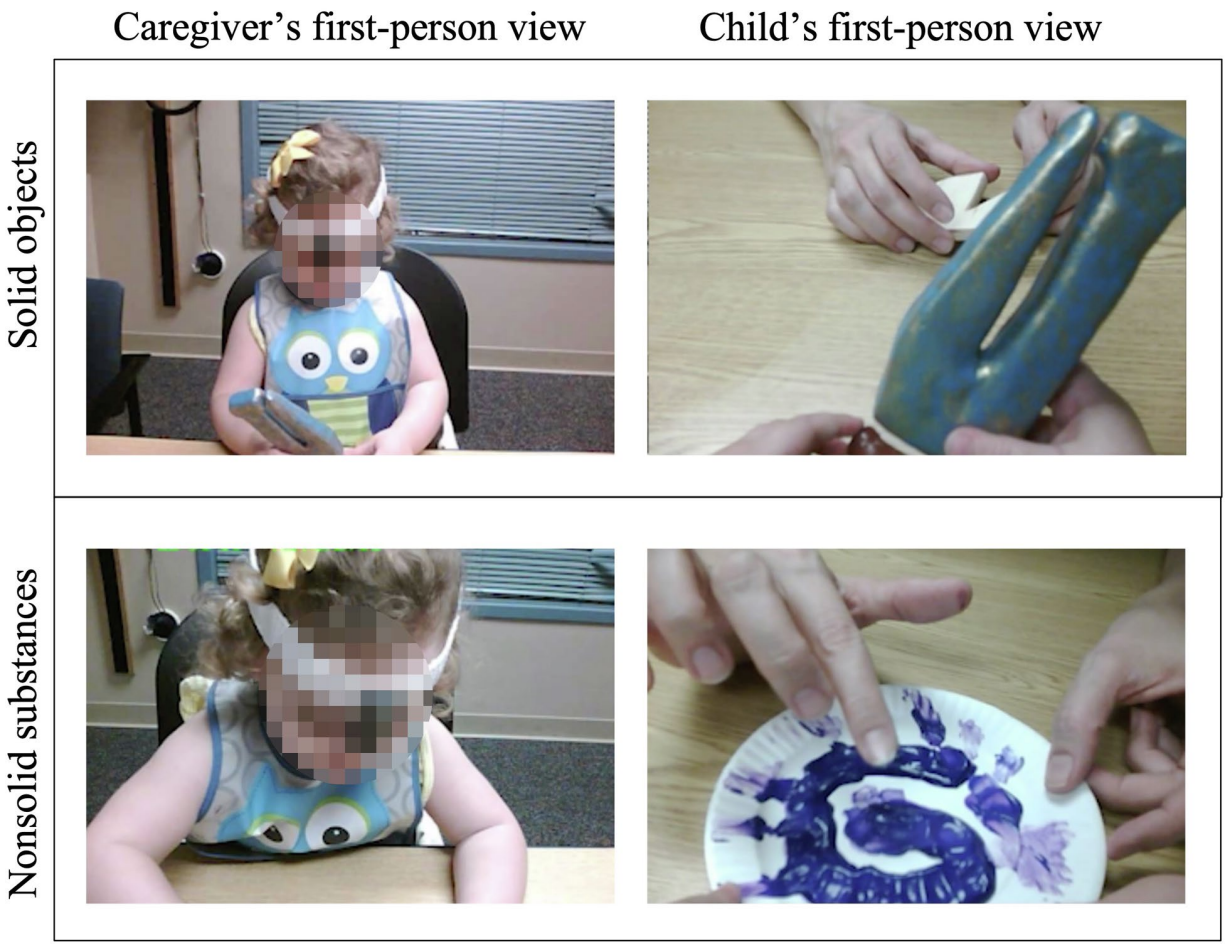
Example first-person views from a caregiver’s (left) and child’s (right) perspectives of free-play with solid objects (top) and nonsolid substances (bottom).

During free-play the child was seated in a booster seat at a table across from their caregiver. Both children and caregivers wore a head camera, a tiny camera centered between the participant’s eyes secured with a headband (see Figure 2). Following procedures similar to those used by Yu and Smith (2012), caregivers were given two sets of three novel exemplars, one set at a time. One set was made up of the three nonsolid exemplars, the other of the three solid exemplars. Order of sets was counterbalanced across dyads. Laminated pictures of each of the objects and their names were velcroed to the caregiver’s side of the table. Caregivers were instructed to “play with your child as you normally would.” They were not told that they need to teach their child the names, but rather that if they choose to name the objects, they should use the names listed on the pictures.

Dyads engaged in free-play with each of the sets for approximately 2 min for a total 4–5 min in free-play. After the experimenter provided the caregiver with one set of stimuli, they left the room and set a timer for 2 min. Following the 2 min of play, the experimenter returned to the room, removed the first set of stimuli, and provided the caregiver with the other set of stimuli, again leaving the room and setting a timer for 2 min. Following those 2 min, the experimenter returned and collected the stimuli to begin the recognition and generalization tasks.

The session was also recorded with two wall-mounted cameras. Prior to free-play, the experimenter clapped to facilitate synchronization of video streams from head and wall-mounted cameras. Videos from each camera were synchronized and combined using Adobe Premiere Pro.

#### Recognition

Immediately following free-play, children completed the recognition task. Participants first completed two warm-up trials (one with solid objects, one with nonsolid substances) to familiarize them to the task. On each warm-up trial, they saw three familiar objects or substances placed on a tray (e.g., duck, car, shoe) and were asked for one (e.g., “Get the duck!”). During warm-up, children were praised for correct choices and re-prompted for incorrect choices. The test trials followed the same procedure with the novel exemplars and without any feedback. On each trial, children saw the three solid exemplars or the three nonsolid exemplars and were asked for one by name (e.g., “Get the bosa!”). This procedure was repeated six times such that each of the exemplars was asked for once, with order and location (left/right/center) counterbalanced. Children’s recognition accuracy (pointing to or picking up the correct object/substance) was coded offline from the wall camera video. One-third of videos were coded for reliability, with >90% agreement.

#### Generalization

Immediately following recognition, children completed the generalization task, used to measure children’s attention to shape/material. Participants first completed two warm-up trials (one with solid objects, one with nonsolid substances). On each warm-up trial, they saw two identical familiar objects or substances (e.g., two identical shoes; two plates of grape jelly arranged in an identical shape), and one distinct familiar object/substance (e.g., a cup; a plate of oatmeal arranged in a distinct shape). The experimenter encouraged the child to touch and explore the objects for approximately 1 min. Then the experimenter held up one of the identical items and set the other stimuli on a tray, saying, e.g., “This is my shoe! Can you get your shoe?” During warm-up, participants received praise for correct choices and were re-prompted for incorrect choices. The six test trials, one for each exemplar, followed the same procedure with novel stimuli and no feedback. On each trial, children saw one of the exemplars from free-play, a novel object/substance identical to the exemplar in shape, but different in material and color (i.e., shape match), and a novel object/substance identical to the exemplar in material, but different in shape and color (i.e., material match). During each trial of the test phase, following exploration, the experimenter held up an exemplar, saying, e.g., “This is my bosa! Can you get your bosa?” Children’s responses (pointing to or picking up the shape or the material match) were coded offline from the wall camera video. One-third of videos were coded for reliability, with >90% agreement.

### Free-Play Coding Procedures

The timing of linguistic, manual, and visual behaviors were coded from synchronized videos using ELAN, a behavioral coding software that allows for accurate coding of event timing (ELAN, 2019). Six of the videos were re-coded for reliability purposes, with a weighted kappa of 0.76, indicating substantial inter-rater reliability.

#### Caregivers’ Linguistic Behaviors

Trained research assistants coded the onset and offset of all caregiver naming events using the audio from free-play session recordings. Naming events were defined as the caregiver’s production of one of the novel names. These events were coded solely on the basis of caregiver speech and separately from children’s manual or visual behaviors.

#### Children’s Manual Behaviors

**Figure 3:**
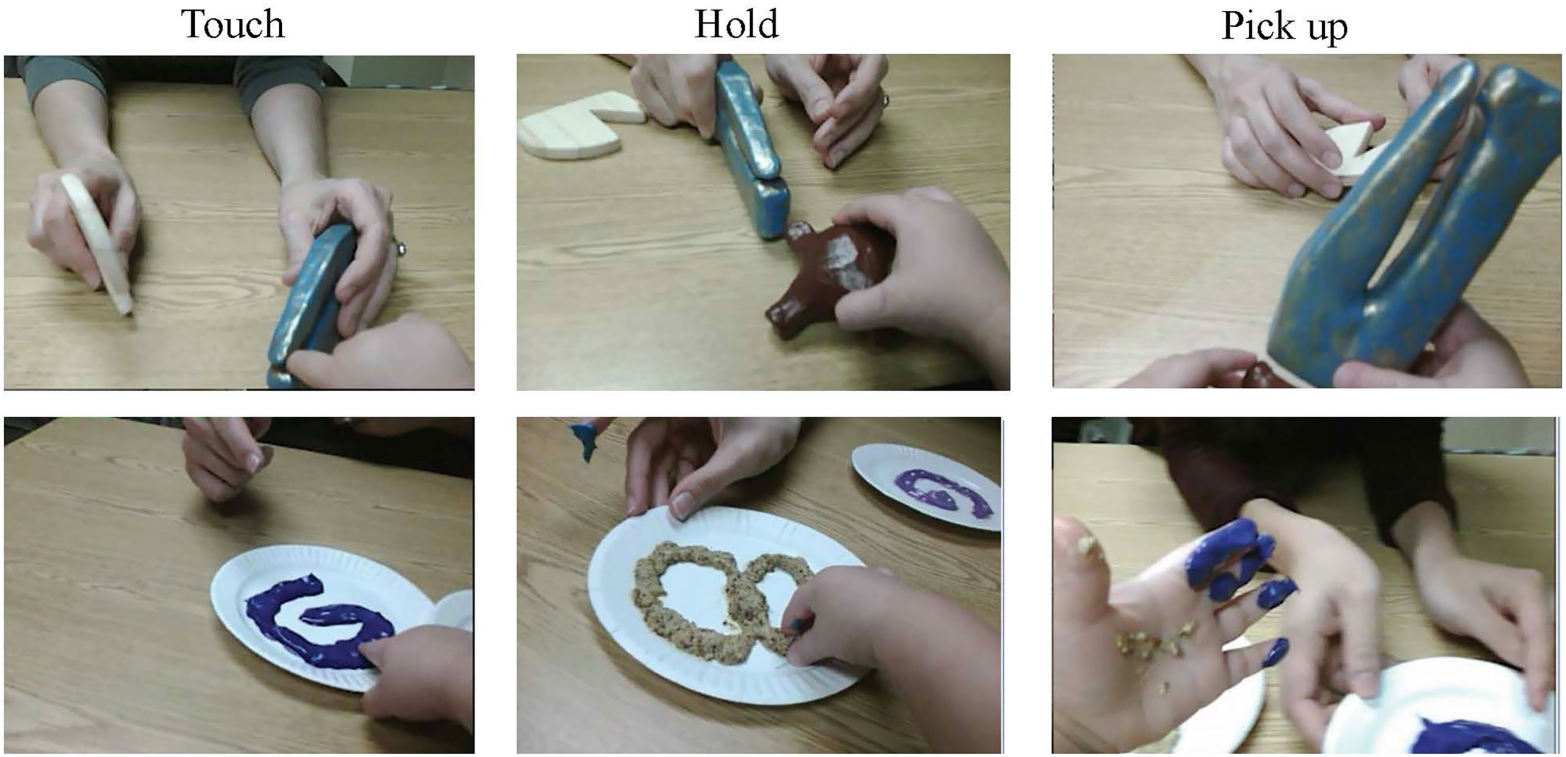
Examples of children’s touch, hold, and pick up behaviors (from left to right) coded for solid (top) and nonsolid (bottom) exemplars during free-play.

Video from the child’s head-camera during free-play was used to code the onset and offset of children’s touching, holding, and pick up behaviors (see Figure 3). *Touching* included instances of the child being in contact with an object/substance while either the table, plate, or caregiver supported the object with fewer than 500 ms of no contact with the object/substance, meaning that instances of rapid poking or tapping were counted as one long instance of touching. *Holding* included instances of the child grasping or scooping (i.e., curling fingers around) the object/substance while their hand, wrist, and/or the object, still was in contact with the table or supported by the caregiver, such that the child was not fully lifting and supporting the object/substance, with no more than 500 ms of non-interaction/attention to the object/substance. *Pick ups* included instances of the child fully supporting the object/substance while their hand, wrist, and object/substance were not in contact with the table or any other surface. If the child brought a nonsolid substance to their mouth and ate it, the pickup ended when their hand was removed from their mouth and the substance was no longer visible/three-dimensional.

The three manual behaviors were coded as mutually exclusive, such that, e.g., a child had to touch an object for at least 500 ms before holding it for both actions to be coded, otherwise only the hold would be coded. In our analyses, we separately examine the total number of manual behaviors a child performed on each object, summing across all three of these behaviors, and the total number of whole-handed actions, summing across holds and pick ups, (*cf.* “messy actions” in Perry et al., 2014).

#### Children’s Visual Behaviors

Video from the child’s head-camera during free-play was used to code the onset and offset of each exemplar being in their child’s view. Codes of which exemplars were in the child’s view were used to assess the coordination, or “overlap,” of children’s manual and visual behaviors and of caregiver and child behaviors as described below. To be coded as in view, an exemplar had to be visible on the video for at least 1,000 ms. The whole exemplars did not have to be fully visible in the video frame to be included as in view as long as enough of the object/substance was visible to determine which exemplar it was. Thus, for nonsolids, a substance had to be three dimensional to be counted (i.e., more than just a smear of color on the child’s hand).

#### Overlapping Behaviors

Following coding, we used ELAN to identify instances of overlap in the timing of linguistic, visual, and manual behaviors. Specifically, we identified (1) the number and duration of instances in which children were looking at and holding or picking up the same object or substance (i.e., “coordinated exploration”); and (2) the number of instances in which caregivers named the object/substance that children were looking at and holding or picking up (i.e., “synchronous naming”).

### Analytic Approach

All data and analysis code are available on the Open Science Framework at https://osf.io/46fv9/. Linear mixed effects regression analyses were conducted in R (R Core Team, 2015) using the *lmer* function, in the “lme4” package (Bates et al., 2014). Chi-square tests comparing models with and without the effect of interest were used to determine significance. All models include random intercepts of subject. Solidity was dummy coded with nonsolid as the reference category.

In our first series of analyses, we used linear mixed effects regression models to compare differences in the number of behaviors that caregivers and children did towards solid objects versus nonsolid substances. We compare the number of instances of caregiver naming, number of overall manual actions, number of whole-handed manual actions, number of instances of coordinated exploration, duration of instances of coordination exploration, rate of coordinated exploration instances out of number of whole-handed actions, number of synchronous naming instances, and rate of synchronous naming instances out of the number of coordinated exploration instances.

In our second series of analyses, we used linear mixed effects regression models to examine associations between free-play behaviors and recognition and generalization test performance. With respect to free-play behaviors, we focused our attention on those overlapping behaviors that have previously been shown to relate to children’s word learning: coordinated exploration (*cf.* “sustained attention” in Yu et al., 2019) and synchronous naming (e.g., Yu and Smith, 2012). In analyses of recognition, we used an approach similar to previous head camera work on novel name recognition (Yu and Smith, 2012), comparing the number of free-play behaviors occurring for recognized names (those that a child accurately selected during recognition) and not recognized names (those that a child did not accurately select during recognition). In generalization analyses we took a similar approach, comparing free-play behaviors for names generalized by shape and names generalized by material. We used separate models for solid and nonsolid stimuli to identify which factors were associated with recognition and generalization for each type of referent. We included child age and vocabulary size (measured by the MCDI) as covariates in these models as these factors are often related to children’s recognition and generalization accuracy (e.g., Kucker et al., 2018).

## Results

### Differences in Free-Play With Solid Objects and Nonsolid Substances

As can be seen in Table 1, children and caregivers performed more of each of the coded behaviors when playing with solid objects than when playing with nonsolid substances. In particular, caregivers were significantly more likely to name the novel solid objects than the novel nonsolid substances, *B =* 1.36, *se* = 0.34, *t =* 3.99, 95% CI [0.69, 2.03]; *X^2^*(1) = 14.52, *p < 0*.001; *d = 0*.96. Children performed more manual actions on solids, *B =* 6.19, *se* = 1.46, *t =* 4.24, 95% CI [3.31, 9.07]; *X^2^*(1) = 16.44, *p < 0*.0001; *d =* 1.02, especially more whole-handed actions, *B =* 7.05, *se* = 1.21, *t =* 5.75, 95% CI [4.65, 9.45]; *X^2^*(1) = 28.48, *p* < 0.00001; *d =* 1.27.

**Table 1:** Descriptive statistics of free-play behaviors (based on each dyad’s average per object of each solidity type).

	Free-play behaviors per object	Solidity	Mean (SD)	Range
Caregiver naming	Number of instances	Solid	2.98 (2.66)	0.00–8.67
		Nonsolid	1.67 (1.59)	0.00–5.67
Child manual exploration	Number of total manual actions	Solid	12.26 (3.57)	6.00–19.33
		Nonsolid	6.07 (3.39)	1.33–13.67
	Number of whole-handed actions	Solid	10.24 (2.80)	5.33–14.00
		Nonsolid	3.19 (2.54)	0.00–7.33
Coordinated exploration	Number of instances	Solid	7.93 (2.28)	5.00–12.33
		Nonsolid	2.74 (2.09)	0.00–6.00
	Ratio of instances to whole-hand actions	Solid	0.77 (0.12)	0.59–0.93
		Nonsolid	0.61 (0.28)	0.00–1.00
	Duration of instances (sec)	Solid	5.04 (2.93)	2.30–12.80
		Nonsolid	5.34 (3.99)	0.00–14.33
Synchronous naming	Number of instances	Solid	1.07 (1.25)	0.00–3.33
		Nonsolid	0.36 (0.50)	0.00–1.33
	Ratio of instances to coordinated exploration instances	Solid	0.19 (0.23)	0.00–0.58
		Nonsolid	0.10 (0.15)	0.00–0.47

Additionally, children were more likely to coordinate visual and manual exploration (i.e., looking at an object/substance while performing whole-handed actions on it) for solid objects than they did for nonsolid substances, *B =* 5.19, *se* = 1.06, *t =* 4.92, 95% CI [3.12, 7.26]; *X^2^*(1) = 21.70, *p* < 0.00001; *d =* 1.09. However, this result is not independent from the finding that children performed more whole-hand actions to solids. Critically, when we examined the ratio of coordinated exploration instances to whole-handed actions (i.e., what proportion of whole-handed actions were done while the child was also looking at the object), we found that the ratio of coordinated exploration instances to whole-handed actions was only marginally higher when children were exploring solid objects than nonsolid substances, *B = 0*.15, *se* = 0.08, *t =* 1.96, 95% CI [−0.0003, 0.31]; *X^2^*(1) = 3.83, *p = 0*.050; *d* = 0.47. Notably, the average *duration* of coordinated exploration instances did not differ for solid objects than nonsolid substances, *X^2^*(1) = 0.06, *p = 0*.812. Together these results demonstrate that although children perform significantly more manual actions towards solid objects, and a slightly higher proportion of their whole-handed actions to solids are coordinated with their looking, the durations of those coordination explorations are similar regardless of the object’s solidity. That coordination exploration has similar durations for solids and nonsolids suggests that it may be more difficult or less motivating for children to coordinate their visual and manual behaviors on nonsolid substances, but once children begin a bout of coordinated exploration they persist for a similar amount of time regardless of what they are exploring.

Finally, we examined differences in caregiver’s synchronous naming. Caregivers were more likely to name the solid objects that children with which were engaged in coordinated exploration (i.e., looking at and performing whole-hand actions upon) than they were for nonsolid substances, *B = 0*.71, *se* = 0.20, *t =* 3.61, 95% CI [0.32, 1.10]; *X^2^*(1) = 12.13, *p = 0*.0005; *d = 0*.87. However, this difference appears to be partially driven by the higher base rate of children’s coordinated exploration of solid objects, as the ratio of synchronous naming instances to coordinated exploration instances was only marginally higher for solid objects than nonsolid substances, *B = 0*.09, *se* = 0.05, *t =* 1.94, 95% CI [−0.001, 0.18]; *X^2^*(1) = 3.73, *p = 0*.054; *d = 0*.47. Critically, there is fairly large variation in the number of free-play behaviors performed by different caregiver-child dyads—especially with respect to the number and rate of synchronous naming instances, with some dyads having 0% and others having as many as over 50% of coordinated explorations being named (see Table 1). We next explore children’s performance in the recognition and generalization tasks to assess how such variation in the free-play task might contribute to differences in children’s word learning.

### Recognition Accuracy and Generalization Performance

**Figure 4:**
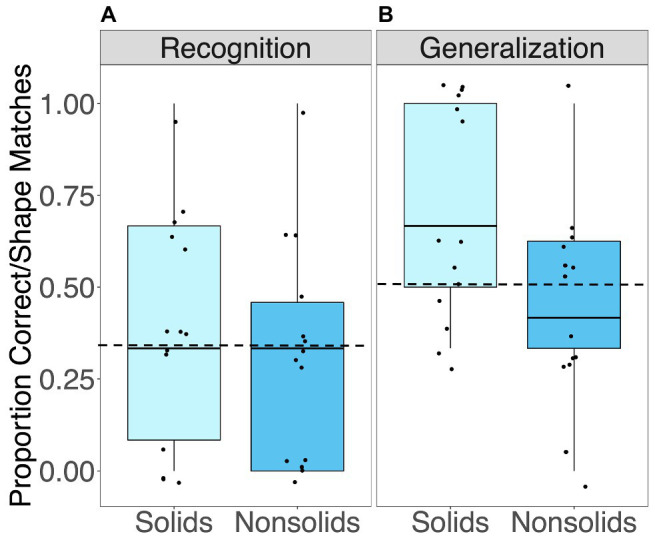
Average **(A)** proportion correct on recognition trials testing children’s associations between novel words and objects, and **(B)** proportion shape matches selected on generalization trials testing children’s attention to shape and material. Dashed lines represent chance (0.33 and 0.50 for the tests of recognition and generalization respectively).

As can be seen in Figure 4A, children’s recognition of novel words was poor, with mean recognition accuracy not differing from chance (0.33) for either solid objects, *M = 0*.38, *t*(13) *= 0*.60, *p* = 0.557; or nonsolid substances, *M = 0*.32, *t*(13) = −0.10, *p = 0*.919. However, as can also be seen in the figure, there was a considerable amount of variability in children’s performance with approximately equal numbers of children showing above chance performance, below chance, and chance performance. In other words, although as a group children showed poor recognition, a sizeable subset of children nevertheless had high levels of recognition accuracy.

As can be seen in Figure 4B, children were not significantly more likely than chance (0.50) to select shape matches when generalizing the names of novel solid objects *M = 0*.63, *t*(13) *=* 1.59, *p* = 0.136. However, half of the children actually did display a shape bias, while the other half were equally likely to select shape and material matches. For nonsolid substances, as a group children were no different from chance in their selection of shape and material matches, *M = 0*.44, *t*(13) *=* −0.84, *p* = 0.418. However, a sizable subset of children (*n* = 7) correctly showed a material bias, generalizing the names of novel substances by similarity in material on most trials.

Notably, the variability in children’s recognition accuracy and generalization performance was quite large. This variability allows us to examine whether individual differences in caregiver-child free-play might have contributed to differences in recognition and generalization. In the next series of analyses, we compare the frequency of each of coordinated exploration and synchronous naming free-play measures for (1) those objects/substances whose names were recognized versus those that were not, and (2) those objects/substances whose names were generalized by similarity in shape versus those generalized by material.

### Associations Between Free-Play Behaviors and Word Learning

#### Recognition Accuracy

**Figure 5:**
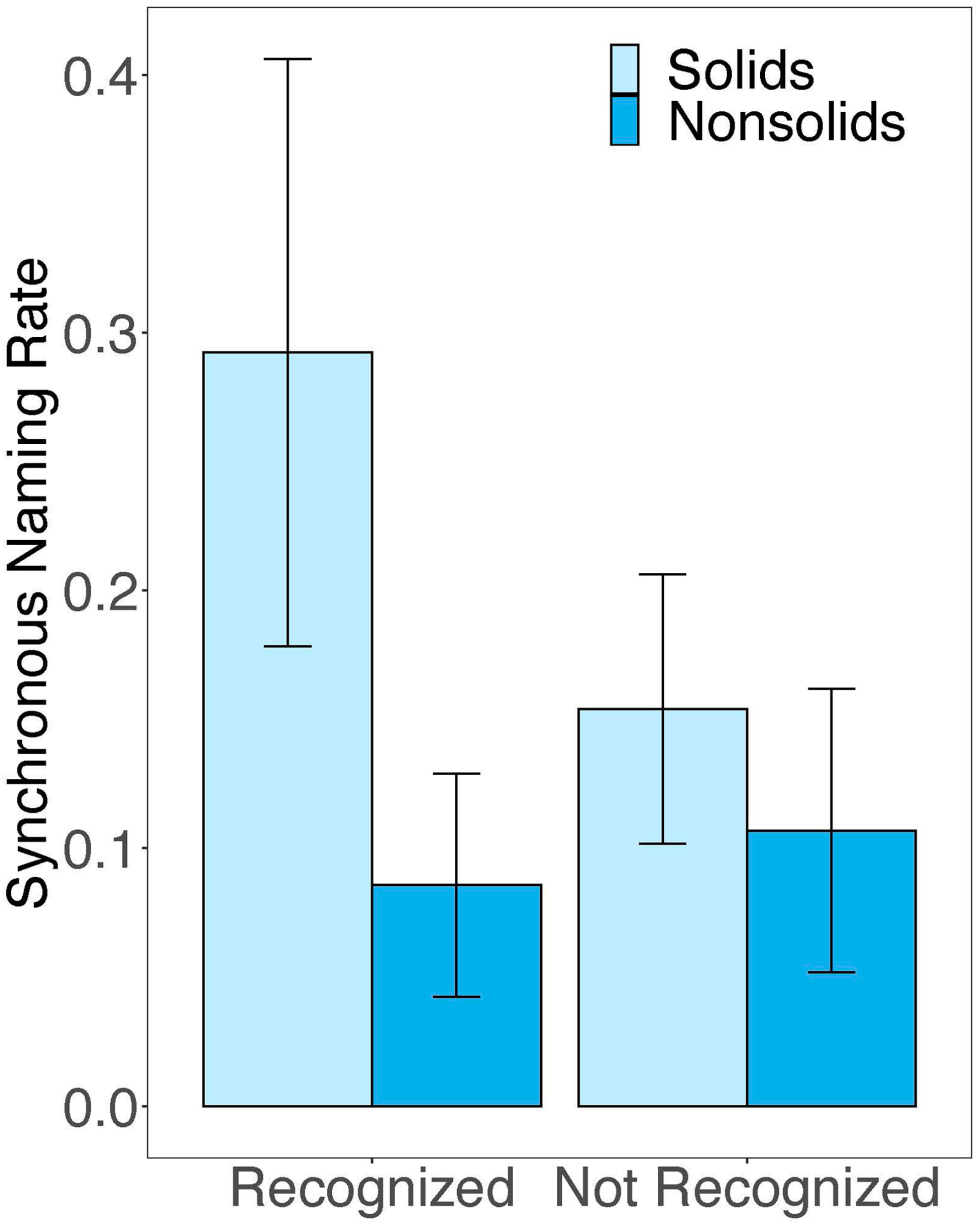
Comparison of synchronous naming during free-play for those novel names that children correctly recognized at test versus those that they did not. Error bars represent standard error of mean.

Using separate models, we compared differences in the number of instances of coordinated exploration and the rate of synchronous naming for solids and nonsolids for novel names that children recognized at test versus those that they did not. Model results are presented in Table 2. As can be seen in Figure 5, rates of synchronous naming during free-play were higher for the names of solid objects that children recognized at test than for those names they did not recognize. However, synchronous naming did not vary with recognition accuracy for the names of nonsolid substances. Additionally, instances of coordinated exploration during free-play were not associated with recognition accuracy for names of solids or nonsolids.

**Table 2:** Results from mixed effects regression analyses comparing differences in free-play behavior related to recognition accuracy.

	Free-play behavior	Predictor	*B*	*se*	*t*	95% CI	Variance	SD	*X^2^*	*p*	*d*
Solid objects	Coordinated exploration	Recognized vs. Not Recognized	−0.47	2.19	−0.21	−4.65, 3.72	–	–	0.05	0.822	0.07
		Age	0.13	0.22	0.57	−0.30, 0.55	–	–	0.36	0.551	0.25
		Vocabulary	−0.007	0.009	−0.77	−0.02, 0.01	–	–	0.65	0.422	
		Subject intercept	–	–	–	–	<0.0001	<0.0001	<0.0001	>0.99	0.18
	Synchronous naming rate	Recognized vs. Not Recognized	0.18	0.08	2.20	0.02, 0.34	–	–	4.94	0.026^*^	0.80
		Age	−0.02	0.01	−1.77	−0.05, 0.001	–	–	3.53	0.060	0.79
		Vocabulary	0.0007	0.0005	1.38	−0.0003, 0.002	–	–	2.25	0.134	0.99
		Subject Intercept	–	–	–	–	0.04	0.19	6.27	0.012	–
Nonsolid substances	Coordinated exploration	Recognized vs. Not Recognized	1.71	1.45	1.18	−1.05, 4.48	–	–	1.53	0.217	0.41
		Age	0.06	0.14	0.45	−0.20, 0.32	–	–	0.24	0.627	0.27
		Vocabulary	−0.004	0.007	−0.68	−0.02, 0.008	–	–	0.51	0.475	0.34
		Subject Intercept	–	–	–	–	0.34	0.59	0.0001	0.992	–
	Synchronous naming rate	Recognized vs. Not Recognized	−0.06	0.07	−0.78	−0.19, 0.08	–	–	0.64	0.424	0.28
		Age	0.0005	0.009	0.06	−0.02, 0.02	–	–	0.003	0.956	0.03
		Vocabulary	0.0003	0.0004	0.83	−0.0004, 0.001	–	–	0.87	0.350	0.43
		Subject Intercept	–	–	–	–	0.01	0.11	1.59	0.207	–

* indicates a significant effect.

#### Generalization Performance

In separate models we compared differences in the number of instances of coordinated exploration and the rate of synchronous naming for solids and nonsolids for novel names that children generalized by similarity in shape versus those that they generalized by similarity in material. Neither rates of synchronous naming nor instances of coordinated exploration during free-play varied with generalization performance (see Table 3).

**Table 3:** Results from mixed effects regression analyses comparing differences in free-play behavior related to generalization (shape vs. material).

	Free-play behavior	Predictor	*B*	*se*	*t*	95% CI	Variance	SD	*X^2^*	*p*	***d***
Solid objects	Coordinated exploration	Shape vs. Material	0.97	1.97	0.49	−2.78, 4.73	–	–	0.27	0.603	0.16
		Age	0.08	0.20	0.41	−0.29, 0.46	–	–	0.38	0.540	0.14
		Vocabulary	−0.005	0.008	−0.58	−0.02, 0.01	–	–	0.19	0.664	0.19
		Subject Intercept	–	–	–	–	<0.0001	<0.0001	<0.0001	>0.99	–
	Synchronous naming rate	Shape vs. Material	−0.07	0.08	−0.88	−0.24, 0.09	–	–	0.82	0.365	0.31
		Age	−0.01	0.01	−1.07	−0.04, 0.01	–	–	1.40	0.237	0.65
		Vocabulary	0.0005	0.0006	0.95	−0.0005, 0.002	–	–	1.23	0.289	0.57
		Subject Intercept	–	–	–	–	0.04	0.20	5.07	0.024	–
Nonsolid substances	Coordinated exploration	Shape vs. Material	−0.18	1.37	−0.13	−2.79, 2.45	–	–	0.02	0.896	0.05
		Age	0.01	0.15	0.07	−0.28, 0.30	–	–	0.07	0.789	0.13
		Vocabulary	−0.002	0.007	−0.25	−0.01, 0.01	–	–	0.007	0.936	0.04
		Subject Intercept	–	–	–	–	0.34	0.58	0.32	0.573	
	Synchronous naming rate	Shape vs. Material	−0.03	0.06	−0.52	−0.16, 0.10	–	–	0.26	0.613	0.20
		Age	−0.002	0.01	−0.17	−0.02, 0.02	–	–	0.03	0.864	0.09
		Vocabulary	0.0004	0.0004	0.87	−0.0004, 0.001	–	–	0.89	0.345	0.45
		Subject Intercept	–	–	–	–	0.01	0.12	1.93	0.165	–

## Discussion

The goal of this study was to compare how caregivers and children manually and visually explore and talk about novel solid objects and nonsolid substances and how those exploratory behaviors relate to recognition and generalization of novel names. We found systematic differences in the frequency and coordination of naming and exploratory behaviors performed on solid objects and nonsolid substances. Children were less likely to manually explore nonsolid substances and caregivers were less likely to name them than they were for solid objects. After controlling for the decrease in manual exploration, we found that the difference in the rate of children’s coordinated of visual and manual exploration was still marginally significant. We also found that caregivers were less likely to synchronize their naming with this coordinated exploration for nonsolids than solids—a difference that was marginally significant even after controlling for differences in children’s base rate of coordinated exploration. Finally, we replicated previous findings that naming synchrony is associated with children’s recognition of the novel names of solid objects. However, we did not find any associations between naming synchrony and recognition of the names of novel nonsolids or with generalization of the names of either solids or nonsolids. Thus, while the results clearly demonstrates that caregivers and children interact with solid objects differently than nonsolid substances during free-play, and the experiences they have with solid objects are associated with recognition of their names, it remains unclear what sort of experiences children actually need to learn the names of *nonsolid substances*. In the following sections we examine possible explanations for the differences in naming and exploration and the lack of associations to nonsolid recognition and generalization.

### Why Do Dyads “Do Less” With Nonsolid Substances?

During free-play, both caregivers (naming) and children (exploration) engaged less with nonsolid substances than they did with solid objects. One likely explanation is that dyads have less prior experience talking about and interacting with nonsolid substances. Indeed, we know by the time the average child is 2.5 years old, they have only learned the names of 14 nonsolid substances (Samuelson and Smith, 1999). The overwhelming majority of this very small list of words are foods and drinks, meaning that children’s opportunities to learn about the names of nonsolid substances may be limited to mealtime contexts. Even at mealtimes, it is possible that children do not have the same opportunities for learning about nonsolids that they do in other contexts for learning about solid objects. For example, consider the rich natural video data from child-worn head camera videos recorded at home recently published by Clerkin et al. (2017). Even though the authors constrained their analysis specifically to mealtimes, naïve coders annotating these videos for the items present in each frame only noted 22 instances of any of the 14 nonsolids listed on the MCDI out of the 24,685 instances of all annotated items,^2^ reflecting a potential overall dearth of experience children have with nonsolids. An additional, but not mutually exclusive, explanation is that the adults coding the videos in the Clerkin study did not perceive nonsolid substances as things to be named, leading to a systematic exclusion of visible substances from their annotations.

Similarly, in our study, it is possible that some caregivers do not perceive nonsolid substances as things to be named, suggesting a developmental history of not naming nonsolids for their child. In the context of our study, this would make the free-play with nonsolids a novel activity for both caregivers and could lead to a lower rate of naming of nonsolids for their child during this task. Alternatively, caregivers may have recognized the particular substances used in our task (e.g., the modi was mayonnaise that had been dyed purple), and this familiarity reduced the stimuli’s salience and contributed to the reduction in caregiver naming of nonsolids. That recognition of nonsolid materials like mayonnaise reduced naming but recognition of solid materials like wood did not, further suggests the possibility that adults have different expectations about naming solids and nonsolids. The idea that adults do not perceive nonsolids as something to be named aligns with Gentner’s natural-partitions hypothesis (see, e.g., Gentner, 1982), in which she proposed that nouns are easier for children to learn than verbs, because many early-learned nouns name concrete objects that are more easily individuated by the perceptual system compared to verbs, which are visually distributed through time as the action they name is performed. Samuelson and Smith (2000) extended this idea to children’s difficulty learning to generalize the names of nonsolid and deformable entities relative to individuatable solid objects. Nonsolids may be more difficult to learn to name because they take on multiple shapes depending on their container or arrangement, whereas solid objects have coherent individuatable shapes to remember. Perhaps this bias in the perceptual system persists throughout development, influencing caregivers and how they may eventually structure interactions with their own children. An interesting direction for future research will be to complement laboratory based tasks with in-home observation or assessments about caregivers’ beliefs about naming and solidity.

Relatedly, the synchrony between caregivers’ naming and children’s coordinated exploration was much lower for free-play with nonsolid substances than with solid objects. Such a low rate of synchronous naming may have prevented us from finding associations to recognition of the names of nonsolids. It may be more difficult for caregivers to coordinate their attention and naming with their child’s attention when playing with nonsolid substances than with solid objects due to both a lack of prior history of doing so and a potential perceptual bias not to think of nonsolids as things to be named.

Finally, an additional, non-mutually exclusive difference between solids and nonsolids is the messiness factor. Although tactile information can be quite informative for identifying the material of nonsolid substances (Lederman and Klatzky, 1990; Perry et al., 2014), obtaining that tactile information can be a messy endeavor, as substances may stick to the child’s hands in trace amounts or even big globs. During free-play, children may have engaged in fewer total instances of manual exploration because they did not want to become messy or they became more interested in wiping the mess off of their hands, preventing them from re-exploring the substances. Indeed, while we found that children engaged in fewer instances of both manual exploration and coordinated exploration for nonsolids, the duration of coordinated exploration events was similar for solids and nonsolids. This pattern potentially suggests that a given instance of engagement in manual-visual behaviors with a nonsolid substance is similar to instances of engagement with solid objects, but that following that engagement with a nonsolid, children might be less likely to re-engage, or perhaps that it was harder for them to initiate engagement with nonsolids in the first place because of their hesitancy to touch them. Notably, such a hesitation could be exacerbated if the child did not have many prior opportunities to engage in such messiness previously, for safety, time, cleanliness, or etiquette concerns the caregiver might have.

### Associations Between Free-Play and Recognition vs. Generalization

Although we replicated prior work finding an association between synchronous naming of solid objects during free-play and recognition of those novel names, synchronous naming of substances was not associated with recognition, and synchronous naming was not associated with children’s generalization of either type of stimuli. In the case of solid objects, why should synchronous naming facilitate the recognition of novel names but not the generalization of those names to other objects of the same shape? Overall, half of the children showed a shape bias, generalizing the names of solid objects by similarity in shape. It is possible that many of the children in the study already had a strong enough bias to attend to shape that they did not require a prolonged free-play exploration with the objects during which the objects were named.

Indeed, the generalization task is set up to allow children to simultaneously compare the exemplar from free-play with the shape match and the material match as they make their decision. Many children in this age range already show a shape bias in such a task without the preceding free-play period. With respect to the nonsolid substances, overall as a group, children performed at chance levels, being equally likely to generalize by shape and material. Such a pattern is also common among children this age, and just as the preceding free-play was not necessary to support a shape bias for solids, it may not have been enough to help support a material bias for nonsolids. These biases instead are built up over developmental time and a prior history of learning individual names and categories (Samuelson, 2002; Smith et al., 2002). Recognition, on the other hand, although also a skill that improves with development and vocabulary acquisition (Bion et al., 2013; Kucker et al., 2018), requires a child to form a strong enough association between a name and referent over time and clearly benefits from extra scaffolding such as caregivers’ synchronous naming (at least for solid objects).

### Limitations and Future Directions

One limitation of the current study was the small sample size. Although common for similar work that obtains relatively large amounts of data per participant (i.e., frame by frame coding of behavior during free-play, multiple recognition trials, and multiple generalization trials), replication with larger samples will be needed to assess generalizability of results and more deeply investigate individual differences in caregiver naming and child exploration. An additional potential limitation was that recognition accuracy was quite poor for many children, especially for the names of nonsolid substances. It is possible that such low accuracy prevented us from detecting meaningful associations between free-play behaviors and recognition. Perhaps more repetition is needed to form a strong enough word-referent association for nonsolids than solids. Another possibility is that perhaps children had formed fragile word-referent associations, but had difficulty with the recognition and generalization tasks, as each task only had one test per new name. Finally, it is also possible that the configuration of nonsolids into distinct shapes—a necessary part of our study design to allow us to probe children’s attention to shape versus material in generalization—made recognition of nonsolids difficult for some reason. Thus, additional directions for future investigation will therefore be to assess exploration and learning over longer periods of time, with repeated test trials, and across different types of stimuli.

### Conclusion

Here we utilized first-person views of children’s and caregivers’ free-play to compare exploration and naming of novel solid objects and nonsolid substances and to examine associations between these behaviors and children’s recognition and generalization of those novel names. Overwhelmingly, both children and caregivers engage in a higher number of exploratory and naming behaviors when playing with solids than with nonsolids. However, although we replicated prior work illustrating the importance of synchronous naming of solid objects in supporting recognition of novel names, it remains unknown what experiences support recognition of the names of novel nonsolid substances. Nevertheless, this work reflects an important first step in identifying the way caregivers influence children’s learning of new words.

## Data Availability Statement

The datasets presented in this study can be found in online repositories. The names of the repository/repositories and accession number(s) can be found at: Open Science Framework: https://osf.io/46fv9.

## Ethics Statement

The studies involving human participants were reviewed and approved by University of Miami Institutional Review Board. Written informed consent to participate in this study was provided by the participants’ legal guardian/next of kin.

## Author Contributions

LP and AV designed the study. LP, AV, SC, and RF collected the data. SC, RF, BG, and AV coded the data. LP processed and analyzed the data. LP, SC, and RF wrote the first draft of the manuscript. All authors contributed to the article and approved the submitted version.

## Funding

This research was funded by a Provost’s Research Award from the University of Miami to LP.

## Conflict of Interest

The authors declare that the research was conducted in the absence of any commercial or financial relationships that could be construed as a potential conflict of interest.

## Publisher’s Note

All claims expressed in this article are solely those of the authors and do not necessarily represent those of their affiliated organizations, or those of the publisher, the editors and the reviewers. Any product that may be evaluated in this article, or claim that may be made by its manufacturer, is not guaranteed or endorsed by the publisher.

## Footnotes

^1^The term “nonsolids” can be used to include a range of stimuli with varying amounts of rigidity, from liquids like water and milk, to substances like hair gel and pudding, to deformables like sponges and towels. Indeed the body of work utilizing looking paradigms often compares children’s expectations for liquids and solid objects (Hespos et al., 2009, 2016), while here, as in other studies utilizing reaching tasks (Soja et al., 1991; Samuelson and Horst, 2007), we use only nonsolid substances that can be arranged in a set shape, as allowing us to pit shape against material in the same way of the solid and nonsolid trials of our forced choice recognition and generalization tasks.

^2^Token counts were calculated from the authors’ shared data files on Databrary (Smith, 2016) using the “Focal” codes in which at least two of the four coders agreed on the presence of an item. The 22 instances were eight frames containing milk, three containing sauce, three containing soda, and eight containing water.

## References

[ref1] Anderson E. M. Hespos S. J. Rips L. J. (2018). Five-month-old infants have expectations for the accumulation of nonsolid substances. Cognition 175, 1–10. doi: 10.1016/j.cognition.2018.02.009, PMID: 29454256

[ref2] Axelsson E. L. Perry L. K. Scott E. J. Horst J. S. (2016). Near or far: The effect of spatial distance and vocabulary knowledge on word learning. Acta Psychol. 163, 81–87. doi: 10.1016/j.actpsy.2015.11.006 26629672

[ref3] Bambach S. Smith L. B. Crandall D. J. Yu C. (2016). Objects in the center: how the infant’s body constrains infant scenes. *2016 Joint IEEE International Conference on Development and Learning and Epigenetic Robotics (ICDL-EpiRob)*, 132–137.

[ref4] Bates D. Mächler M. Bolker B. Walker S. (2014). Fitting linear mixed-effects models using lme4. J. Stat. Softw. 67, 1–48. doi: 10.48550/arXiv.1406.5823

[ref5] Benitez V. L. Smith L. B. (2012). Predictable locations aid early object name learning. Cognition 125, 339–352. doi: 10.1016/j.cognition.2012.08.006, PMID: 22989872 PMC3472129

[ref6] Bergelson E. Aslin R. N. (2017). Nature and origins of the lexicon in 6-mo-olds. Proc. Natl. Acad. Sci. 114, 12916–12921. doi: 10.1073/pnas.1712966114, PMID: 29158399 PMC5724273

[ref7] Biederman I. (1995). Visual Object Recognition. Cambridge, MA: MIT Press.

[ref8] Bion R. A. H. Borovsky A. Fernald A. (2013). Fast mapping, slow learning: disambiguation of novel word–object mappings in relation to vocabulary learning at 18, 24, and 30 months. Cognition 126, 39–53. doi: 10.1016/j.cognition.2012.08.008, PMID: 23063233 PMC6590692

[ref9] Bruner J. S. (1975). The ontogenesis of speech acts. J. Child Lang. 2, 1–19. doi: 10.1017/S0305000900000866

[ref10] Carpenter M. Nagell K. Tomasello M. Butterworth G. Moore C. (1998). Social cognition, joint attention, and communicative competence from 9 to 15 months of age. Monogr. Soc. Res. Child Dev. 63:174. doi: 10.2307/1166214 9835078

[ref11] Chen C. Castellanos I. Yu C. Houston D. M. (2020). What leads to coordinated attention in parent–toddler interactions? Children’s Hearing Status Matters. Dev. Sci. 23:e12919. doi: 10.1111/desc.12919, PMID: 31680414 PMC7160036

[ref12] Clerkin E. M. Hart E. Rehg J. M. Yu C. Smith L. B. (2017). Real-world visual statistics and infants’ first-learned object names. Philos. Trans. R. Soc. B Biol. Sci. 372:20160055. doi: 10.1098/rstb.2016.0055, PMID: 27872373 PMC5124080

[ref13] Custode S. A. Tamis-LeMonda C. (2020). Cracking the code: social and contextual cues to language input in the home environment. Infancy 25, 809–826. doi: 10.1111/infa.12361, PMID: 32869471

[ref14] Dale P. S. Fenson L. (1996). Lexical development norms for young children. Behav. Res. Methods Instrum. Comput. 28, 125–127. doi: 10.3758/BF03203646

[ref15] DeBolt M. C. Rhemtulla M. Oakes L. M. (2020). Robust data and power in infant research: A case study of the effect of number of infants and number of trials in visual preference procedures. Infancy 25, 393–419. doi: 10.1111/infa.12337, PMID: 32744759

[ref16] Denham S. A. Mitchell-Copeland J. Strandberg K. Auerbach S. Blair K. (1997). Parental contributions to preschoolers’ emotional competence: direct and indirect effects. Motiv. Emot. 21, 65–86. doi: 10.1023/A:1024426431247

[ref17] ELAN (2019). ELAN - The Language Archive (5.6) (Computer software). Max Planck Institute for Psycholinguistics. Available at: https://tla.mpi.nl/tools/tla-tools/elan/citing_elan/

[ref18] Fenson L. Dale P. S. Reznick J. S. Bates E. Thal D. J. Pethick S. J.. (1994). Variability in early communicative development. Monogr. Soc. Res. Child Dev. 59, i–185. doi: 10.2307/1166093 7845413

[ref19] Gentner D. (1982). “Why nouns are learned before verbs: linguistic relativity versus natural partitioning” in Language Development: Language, Thought, and Culture. ed. Kuczaj S. A. (New Jersey: Erlbaum), 301–334.

[ref20] Gillette J. Gleitman H. Gleitman L. Lederer A. (1999). Human simulations of vocabulary learning. *Vol 2.* Cognition 73, 135–176. doi: 10.1016/S0010-0277(99)00036-0 10580161

[ref21] Golinkoff R. M. Can D. D. Soderstrom M. Hirsh-Pasek K. (2015). (Baby)talk to me: The social context of infant-directed speech and its effects on early language acquisition. Curr. Dir. Psychol. Sci. 24, 339–344. doi: 10.1177/0963721415595345

[ref22] Hammond S. I. Müller U. Carpendale J. I. M. Bibok M. B. Liebermann-Finestone D. P. (2012). The effects of parental scaffolding on preschoolers’ executive function. Dev. Psychol. 48, 271–281. doi: 10.1037/a0025519, PMID: 21928877

[ref23] Hespos S. J. Ferry A. L. Anderson E. M. Hollenbeck E. N. Rips L. J. (2016). Five-month-old infants have general knowledge of how nonsolid substances behave and interact. Psychol. Sci. 27, 244–256. doi: 10.1177/0956797615617897, PMID: 26744069

[ref24] Hespos S. J. Ferry A. L. Rips L. J. (2009). Five-month-old infants have different expectations for solids and liquids. Psychol. Sci. 20, 603–611. doi: 10.1111/j.1467-9280.2009.02331.x, PMID: 19368696

[ref25] Huntley-Fenner G. Carey S. Solimando A. (2002). Objects are individuals but stuff doesn’t count: perceived rigidity and cohesiveness influence infants’ representations of small groups of discrete entities. Cognition 85, 203–221. doi: 10.1016/S0010-0277(02)00088-4, PMID: 12169409

[ref26] Kucker S. C. McMurray B. Samuelson L. K. (2018). Too much of a good thing: how novelty biases and vocabulary influence known and novel referent selection in 18-month-old children and associative learning models. Cogn. Sci. 42, 463–493. doi: 10.1111/cogs.12610, PMID: 29630722 PMC5980730

[ref27] Landau B. Smith L. B. Jones S. S. (1988). The importance of shape in early lexical learning. Cogn. Dev. 3, 299–321. doi: 10.1016/0885-2014(88)90014-7

[ref28] Lederman S. J. Klatzky R. L. (1990). Haptic classification of common objects: knowledge-driven exploration. Cogn. Psychol. 22, 421–459. doi: 10.1016/0010-0285(90)90009-S, PMID: 2253454

[ref29] Markman E. M. (1991). “The whole-object, taxonomic, and mutual exclusivity assumptions as initial constraints on word meanings” in Perspectives on Language and Thoughts: Interrelations in Development. eds. Byrnes J. P. Gelman S. A. (London: Cambridge University Press), 72–106.

[ref30] McQuillan M. E. Smith L. B. Yu C. Bates J. E. (2020). Parents influence the visual learning environment Through Children’s manual actions. Child Dev. 91, e701–e720. doi: 10.1111/cdev.13274, PMID: 31243763 PMC6930973

[ref31] Pereira A. F. James K. H. Jones S. S. Smith L. B. (2010). Early biases and developmental changes in self-generated object views. J. Vis. 10:22. doi: 10.1167/10.11.22, PMID: 20884517 PMC3049954

[ref32] Perry L. K. Axelsson E. L. Horst J. S. (2016). Learning what to remember: vocabulary knowledge and Children’s memory for object names and features. Infant Child Dev. 25, 247–258. doi: 10.1002/icd.1933

[ref33] Perry L. K. Custode S. A. Fasano R. M. Gonzalez B. M. Savy J. D. (2021). What is the buzz About iconicity? How iconicity in caregiver speech supports Children’s word learning. Cogn. Sci. 45:e12976. doi: 10.1111/cogs.12976, PMID: 33873243

[ref34] Perry L. K. Kucker S. C. (2019). The heterogeneity of word learning biases in late talking children. J. Speech Lang. Hear. Res. 62, 554–563. doi: 10.1044/2019_JSLHR-L-ASTM-18-0234, PMID: 30950748

[ref35] Perry L. K. Saffran J. R. (2017). Is a pink cow still a cow? Individual differences in toddlers’ vocabulary knowledge and lexical representations. Cogn. Sci. 41, 1090–1105. doi: 10.1111/cogs.12370, PMID: 27059812 PMC5052107

[ref36] Perry L. K. Samuelson L. K. (2011). The shape of the vocabulary predicts the shape of the Bias. Front. Psychol. 2:345. doi: 10.3389/fpsyg.2011.00345, PMID: 22125547 PMC3222225

[ref37] Perry L. K. Samuelson L. K. Burdinie J. B. (2014). Highchair philosophers: The impact of seating context-dependent exploration on children’s naming biases. Dev. Sci. 17, 757–765. doi: 10.1111/desc.12147, PMID: 24289734 PMC4040339

[ref38] Perry L. K. Samuelson L. K. Malloy L. M. Schiffer R. N. (2010). Learn locally, think globally: exemplar variability supports higher-order generalization and word learning. Psychol. Sci. 21, 1894–1902. doi: 10.1177/0956797610389189, PMID: 21106892 PMC3144952

[ref39] Quine W. V. O. (1960). Word and Object. Cambridge, MA: MIT Press.

[ref40] R Core Team (2015). R: A language and environment for statistical computing.

[ref41] Rips L. J. Hespos S. J. (2015). Divisions of the physical world: concepts of objects and substances. Psychol. Bull. 141, 786–811. doi: 10.1037/bul0000011, PMID: 25822132

[ref42] Rips L. J. Hespos S. J. (2019). Concepts of objects and substances in language. Psychon. Bull. Rev. 26, 1238–1256. doi: 10.3758/s13423-019-01613-w, PMID: 31197757

[ref43] Samuelson L. K. (2002). Statistical regularities in vocabulary guide language Acquisition in Connectionist Models and 15-20-month-olds. Dev. Psychol. 38, 1016–1037. doi: 10.1037/0012-1649.38.6.1016, PMID: 12428712

[ref44] Samuelson L. K. Horst J. S. (2007). Dynamic noun generalization: moment-to-moment interactions shape Children’s naming biases. Infancy 11, 97–110. doi: 10.1207/s15327078in1101_5

[ref45] Samuelson L. K. Smith L. B. (1999). Early noun vocabularies: do ontology, category structure and syntax correspond? Cognition 73, 1–33. doi: 10.1016/S0010-0277(99)00034-7, PMID: 10536222

[ref46] Samuelson L. K. Smith L. B. (2000). Children’s attention to rigid and deformable shape in naming and non-naming tasks. Child Dev. 71, 1555–1570. doi: 10.1111/1467-8624.00248, PMID: 11194256

[ref47] Samuelson L. K. Smith L. B. Perry L. K. Spencer J. P. (2011). Grounding word learning in space. PLoS One 6:e28095. doi: 10.1371/journal.pone.0028095, PMID: 22194807 PMC3237424

[ref48] Slone L. K. Sandhofer C. M. (2017). Consider the category: the effect of spacing depends on individual learning histories. J. Exp. Child Psychol. 159, 34–49. doi: 10.1016/j.jecp.2017.01.010, PMID: 28266333 PMC5393938

[ref49] Smith L. B. (2016). Real-world visual statistics and infants’ first-learned object names. Databrary. Available at: 10.17910/B7.268 (Accessed July 20, 2021). PMC5124080 27872373

[ref50] Smith L. B. Jayaraman S. Clerkin E. Yu C. (2018). The developing infant creates a curriculum for statistical learning. Trends Cogn. Sci. 22, 325–336. doi: 10.1016/j.tics.2018.02.004, PMID: 29519675 PMC5866780

[ref51] Smith L. B. Jones S. S. Landau B. Gershkoff-Stowe L. Samuelson L. (2002). Object name learning provides on-the-job training for attention. Psychol. Sci. 13, 13–19. doi: 10.1111/1467-9280.00403, PMID: 11892773

[ref52] Smith L. B. Yu C. Pereira A. F. (2011). Not your mother’s view: The dynamics of toddler visual experience. Dev. Sci. 14, 9–17. doi: 10.1111/j.1467-7687.2009.00947.x, PMID: 21159083 PMC3050020

[ref53] Smith L. B. Yu C. Yoshida H. Fausey C. M. (2015). Contributions of head-mounted cameras to studying the visual environments of infants and young children. J. Cogn. Dev. 16, 407–419. doi: 10.1080/15248372.2014.933430, PMID: 26257584 PMC4527180

[ref54] Soja N. N. (1992). Inferences about the meanings of nouns: The relationship between perception and syntax. Cogn. Dev. 7, 29–45. doi: 10.1016/0885-2014(92)90003-A

[ref55] Soja N. N. Carey S. Spelke E. S. (1991). Ontological categories guide young children’s inductions of word meaning: object terms and substance terms. Cognition 38, 179–211. doi: 10.1016/0010-0277(91)90051-5, PMID: 2049905

[ref56] Soska K. C. Adolph K. E. Johnson S. P. (2010). Systems in development: motor skill acquisition facilitates three-dimensional object completion. Dev. Psychol. 46, 129–138. doi: 10.1037/a0014618, PMID: 20053012 PMC2805173

[ref57] Suanda S. H. Barnhart M. Smith L. B. Yu C. (2019). The signal in the noise: The visual ecology of parents’ object naming. Infancy 24, 455–476. doi: 10.1111/infa.12278, PMID: 31551663 PMC6759226

[ref58] Subrahmanyam K. Landau B. Gelman R. (1999). Shape, material, and syntax: interacting forces in Children’s learning in novel words for objects and substances. Lag. Cogn. Proc. 14, 249–281. doi: 10.1080/016909699386301

[ref59] Tamis-LeMonda C. S. Custode S. Kuchirko Y. Escobar K. Lo T. (2019). Routine language: speech directed to infants During home activities. Child Dev. 90, 2135–2152. doi: 10.1111/cdev.13089, PMID: 29766498

[ref60] Tamis-LeMonda C. S. Kuchirko Y. Song L. (2014). Why is infant language learning facilitated by parental responsiveness? Curr. Dir. Psychol. Sci. 23, 121–126. doi: 10.1177/0963721414522813

[ref61] vanMarle K. Scholl B. J. (2003). Attentive tracking of objects versus substances. Psychol. Sci. 14, 498–504. doi: 10.1111/1467-9280.03451, PMID: 12930483

[ref62] Vygotsky L. (1987). “The genesis of higher Mental Functions,” in The History of the Development of Higher mental functions. Vol. 4 ed. Rieber R. W., (New York: Plennum), 97–120.

[ref63] Yoshida H. Smith L. B. (2008). What’s in view for toddlers? Using a head camera to study visual experience. Infancy 13, 229–248. doi: 10.1080/15250000802004437, PMID: 20585411 PMC2888512

[ref64] Yu C. Smith L. B. (2012). Embodied attention and word learning by toddlers. Cognition 125, 244–262. doi: 10.1016/j.cognition.2012.06.016, PMID: 22878116 PMC3829203

[ref65] Yu C. Smith L. B. (2013). Joint attention without gaze following: human infants and their parents coordinate visual attention to objects through eye-hand coordination. PLoS One 8:e79659. doi: 10.1371/journal.pone.0079659, PMID: 24236151 PMC3827436

[ref66] Yu C. Suanda S. H. Smith L. B. (2019). Infant sustained attention but not joint attention to objects at 9 months predicts vocabulary at 12 and 15 months. Dev. Sci. 22:e12735. doi: 10.1111/desc.12735, PMID: 30255968 PMC6918481

[ref67] Yurovsky D. Smith L. B. Yu C. (2013). Statistical word learning at scale: The baby’s view is better. Dev. Sci. 16, 959–966. doi: 10.1111/desc.12036, PMID: 24118720 PMC4443688

